# The Relationship Between Laboratory Parameters and Coronary Slow Flow

**DOI:** 10.3390/jcm14238477

**Published:** 2025-11-29

**Authors:** Muhammet Cakas, Hayrullah Yurdakul, Seda Elcim Yildirim, Tarik Yildirim, Bahadir Caglar, Suha Serin

**Affiliations:** 1 Genc City Hospital, 12000 Bingol, Turkey; muhammetcakas@gmail.com; 2 Nizip City Hospital, 27300 Gaziantep, Turkey; h.yurdakul@outlook.com; 3 Department of Cardiology, Faculty of Medicine, Balikesir University, 10900 Balikesir, Turkey; sedaelcimdurusoy@gmail.com (S.E.Y.); kdrtarik@gmail.com (T.Y.); 4 Department of Emergency Medicine, Faculty of Medicine, Balikesir University, 10900 Balikesir, Turkey; suhaserin@gmail.com

**Keywords:** chest pain, coronary slow flow, inflammation

## Abstract

**Background/Objectives**: One of the most prevalent reasons for attending the emergency department (ED) is chest pain. There are many causes of its etiology. Recent studies indicate that coronary slow flow should be considered not only an angiographic phenomenon but also a clinical syndrome. In our study, we aimed to identify laboratory parameters indicative of coronary slow flow. **Methods**: Patients who presented to the Emergency Department of Balikesir University Hospital with chest pain and underwent coronary angiography between 2019 and 2023 were evaluated. A group of 107 patients with primary coronary slow flow was included as the patient group, while 108 patients without any pathology were included as the control group. Demographic, laboratory, clinical, and angiographic parameters were compared between the two groups to determine the predictors of coronary slow flow. **Results**: In our study, RCA dominance was detected in the control group, while Cx dominance was detected in our patient group (*p* < 0.001). CRP and the CRP/albumin ratio were observed to be higher in the patient group (*p* < 0.001). **Conclusions**: The inflammatory markers CRP and CRP/albumin ratio were found to be statistically significantly higher in the patient group. These parameters can be used to predict coronary slow flow in the emergency department.

## 1. Introduction

The management of patients with chest pain, one of the most common reasons for emergency department visits, is complex and demanding [1]. This situation leads to the inefficient use of emergency department resources and delays in diagnosis. In managing patients with chest pain in the emergency department, it is critical to rapidly identify the few with acute coronary syndrome (ACS) or other cardiac causes among the many with non-life-threatening, predominantly noncardiac chest pain [2].

Diagnostic tools such as electrocardiogram, cardiac biomarkers, and bedside echocardiography are the primary diagnostic methods used to distinguish low-risk patients from those with ACS. However, none of these methods alone can rule out ACS. Clinical risk scores (HEART, TIMI, SVEAT, etc.) have been developed to more easily identify cardiac-related chest pain [3].

According to commonly used scoring systems, patients presenting with cardiac chest pain and a negative electrocardiogram (ECG) for acute coronary syndrome (ACS), but who have risk factors such as hypercholesterolemia, hypertension, diabetes or smoking are considered to be at moderate risk. These patients undergo more diagnostic tests to make a diagnosis [4].

Coronary angiography (CAG), one of the advanced diagnostic tests, is the best method for detecting coronary stenosis. Coronary slow flow (CSF) is an indicator of delayed filling of the terminal vessels of the coronary arteries in the absence of coronary stenosis on CAG [5].

Studies have shown that 80–90% of patients with CSF experience chest pain, and 33% of these patients require repeated hospitalizations. This significantly reduces the quality of life of patients with CSF and increases the burden on the healthcare system [6]. Approximately 2.5% of these patients have a poor prognosis, such as sudden death or life-threatening, serious ventricular arrhythmias [7]. Therefore, patients with CSF should receive early diagnosis and be monitored at regular intervals.

In our study, we aimed to identify parameters that could predict CSF in patients presenting to the emergency department with chest pain.

## 2. Materials and Methods

### 2.1. Selection of Study Groups and Study Design

This study is a single-center, observational, and retrospective study. It was conducted with the approval of the Ethics Committee of Balikesir University. Patients presenting to the Emergency Department of Balikesir University Hospital with chest pain or angina-equivalent symptoms and underwent coronary angiography between 2019 and 2023 were evaluated. Following coronary angiography performed by cardiologists, 107 patients with primary coronary slow flow were included in the patient group, while 108 patients with normal coronary arteries and no pathology were included in the control group. Patient data and angiography images were accessed through the hospital information management system.

The following criteria were used to determine exclusion from the study:

- Those below the age of 18.
- Those diagnosed with ST-elevation myocardial infarction.
- Patients who had previously undergone coronary revascularization (CABG-O and PTCA stent).
- Patients receiving immunosuppressive therapy.
- Patients with renal insufficiency or those undergoing routine dialysis treatment.
- Patients with liver failure (aspartate transaminase (AST) and alanine transaminase (ALT) levels > 3 times the normal value).
- Patients with gout or those receiving hypouricemic drug therapy.
- Patients with a known history of malignancy.
- Patients with a history of cardiomyopathy (restrictive, hypertrophic, and dilated).

### 2.2. Coronary Angiographic Evaluation

Coronary angiography procedures were performed using the standard Judkins technique with a Siemens imaging system via the femoral or radial percutaneous route. Angiographic images were obtained in the left and right oblique planes at caudal and cranial angles in order to demonstrate the coronary arteries. Images were obtained at a film speed of 30 frames per second (30 fps). In all cases, a nonionic low-osmolar contrast medium (Iohexol, Omnipol 300 mg I/mL; Polifarma, Istanbul, Turkey) was utilized. Coronary angiography images were reviewed with a cardiologist, and the dominant artery was identified and recorded.

Coronary slow flow was determined using the TIMI frame count (TFC) defined by Gibson and colleagues. The TFC is the most commonly used method in the literature. The TIMI frame count is determined as the number of “sineframes” required for the contrast agent to pass to the distal end of the left anterior descending coronary artery, with the aim of more objectively evaluating coronary blood flow as a continuous numerical variable. “The first frame” is defined as the point at which the contrast agent enters the coronary artery for the first time during measurement. The last frame in which the contrast medium reaches the distal end of the left anterior descending coronary artery is defined as the “final frame.” TFC is then calculated by taking the difference between these two frames. This method allows coronary flow to be expressed as a numerical value and provides a reliable and globally accepted standardization of CSF [8].

The frames at which the contrast agent first enters the coronary artery and reaches the distal turning point are determined as the first and last frames, respectively. The initial frame is captured when greater than 70% of the coronary vessel lumen becomes opacified through antegrade filling. The distal markers for the final frames were defined as follows: the distal bifurcation, also known as the “whale tail”, for the left anterior descending (LAD) artery; the most distal bifurcation of the broad marginal branch for the circumflex (Cx) artery; and the first branch of the posterolateral segment.

The cutoff values for TFC for the normal filling of the epicardial coronary arteries were 22.1 ± 4.1 for the Cx artery and 20.4 ± 3.1 for the right coronary artery (RCA) [9,10]. Given the generally longer course of the LAD artery in comparison to that of the right RCA and Cx arteries, TFC was divided by 1.7 for the LAD artery, in order to obtain the corrected TFC (dTFC) value. The dTFC for the LAD artery was thus determined to be 21.1 ± 1.5. The mean TFC value was calculated from the mean values of the square counts of the RCA, LAD, and Cx arteries. Any TFC value above two standard deviations from these published thresholds (TFC < 2) was considered a diagnosis of “coronary slow flow”.

### 2.3. Laboratory Tests

The first samples of blood were taken from the patients within 10 min of them arriving at the emergency department with chest pain. Venous blood samples were taken again from hospitalized patients after 12 h of fasting and evaluated.

Blood samples were analyzed using EDTA-containing tubes for hematology parameters (Beckman Coulter DXH-800; Brea, CA, USA), gel-containing tubes for biochemistry parameters (Beckman Coulter AU680; Brea, CA, USA), and the nephelometry technique for C-reactive protein (CRP) values (Siemens, Munich, Germany, BN II).

The calculation of the CRP/albumin ratio was performed through the division of the CRP value by the albumin value. The calculation of the uric acid/albumin ratio was performed through the division of the uric acid value by the albumin value. In addition, triglyceride (TG)/glucose indices were calculated using the formula “Ln [TG (fasting) (mg/dL) × glucose (fasting) (mg/dL)/2]”.

Patients’ plasma atherogenic index values were also calculated and recorded using the formula “[log (TG/high-density lipoprotein (HDL))]” Total cholesterol/HDL values were calculated to determine the Castelli Risk Index-1, and low-density lipoprotein (LDL)/HDL ratios were calculated to determine the Castelli Risk Score-2 indices.

### 2.4. Statistical Analysis

SPSS version 20.0 software (IBM Inc., Chicago, IL, USA) was used for all analyses. The Kolmogorov–Smirnov test was used to assess the normality of the distribution of continuous variables. Continuous variables are presented as mean ± standard deviation or median, minimum, and maximum values according to their distribution shape. Categorical variables are presented as numbers and percentages. The chi-square and Fisher’s exact tests were used to compare categorical variables. In order to compare continuous variables with a normal distribution, the independent samples *t*-test was employed. By contrast, for continuous variables lacking a normal distribution, the Mann–Whitney U test was utilized for comparison. All analyses were two-tailed, and *p* < 0.05 was considered statistically significant.

## 3. Results

**Figure 1:**
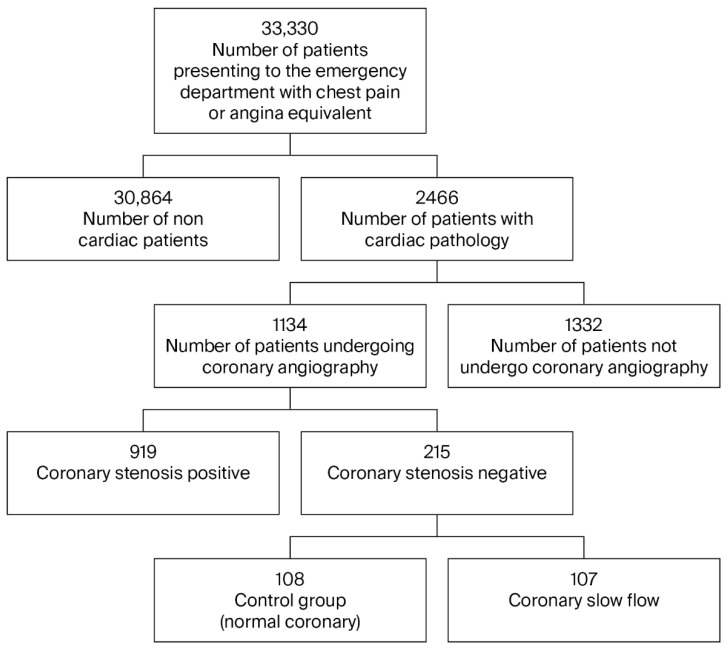
Patient flow diagram.

Over the 5-year study period (2019–2023), 33,330 patients who presented to the university hospital emergency department with chest pain were examined. As a result of the examinations, 1134 patients underwent CAG. In total, 215 patients with no stenosis detected on CAG were evaluated (Figure 1); 107 patients with CSF were assigned to the study group, and the other 108 patients were designated the control group. An analysis of the data revealed no statistically significant differences between the patient group and the control group with regard to age, gender, diabetes, hypertension, and hyperlipidemia (Table 1). When evaluated in terms of coronary artery dominance, RCA dominance was identified in the control group, and Cx dominance was identified in the patient group (*p* < 0.001).

The CRP value was found to be significantly higher in the patient group (*p* < 0.001). The laboratory data for both groups are shown in Table 2.

In recent years, researchers studying predictors of cardiac risk have used indices such as the “CRP/albumin ratio” and “serum uric acid/albumin ratio” as markers of inflammation, the “plasma atherogenic index” as a marker of subclinical atherosclerosis, the “triglyceride/glucose index” as an indicator of insulin resistance, and the “Castelli 1 and Castelli 2 risk indices” [11,12] (Table 3).

## 4. Discussion

Chest pain is one of the most common reasons for admission to the emergency department (ED). Although initial evaluations aim to rule out life-threatening cardiac conditions, definitive diagnoses often require further testing, resulting in prolonged stays in the ED. CSF is frequently observed in this patient group, yet it is often overlooked. Previous studies have shown that 80–90% of patients with CSF experience chest pain and that approximately one-third require recurrent hospitalization. Furthermore, CSF has been linked to serious complications, including malignant ventricular arrhythmias and sudden cardiac death.

In our study, a significant difference in coronary dominance was observed between groups. RCA dominance was more prevalent in the control group, whereas Cx dominance was significantly higher in the CSF group. This finding suggests a potential association between coronary dominance and coronary flow patterns, providing a valuable addition to the existing literature. Although there is insufficient literature on this subject, unlike our study, it has been observed that the incidence of CSF is lower in left-dominant coronary systems due to the wide distribution area of Cx and the presence of a developed vascular field in this region. In patients with right dominance, CSF is reported more frequently due to the RCA supplying a large area and the frequent occurrence of endothelial dysfunction [13,14].

While the exact pathophysiology of CSF remains unclear, inflammation is recognized as a key contributing factor [15,16]. In our analysis, CRP levels and the CRP/albumin ratio—both of which are inflammatory markers—were significantly higher in the CSF group [17,18]. The CRP/albumin ratio reflects the balance between positive and negative acute-phase reactants and is considered a more robust indicator of inflammation than either parameter alone. Our results support this perspective and are consistent with previous reports.

Other proposed mechanisms involved in the development of CSF include endothelial dysfunction, microvascular impairment, and subclinical atherosclerosis [19]. However, our study found no significant differences between groups in terms of the plasma atherogenic index, the triglyceride/glucose index (TGI), or the Castelli risk indices. Although prior research has demonstrated associations between these markers and CSF, the acute nature of our study population may have impacted these outcomes. Further research involving patients at different stages of the disease is needed to clarify these associations.

In patients with CSF, increased levels of inflammatory biomarkers and Endothelin-1 (ET-1), and decreased levels of nitric oxide (NO), have been observed. The decrease in NO and the increase in ET-1 are the causative factors in microvascular vasoconstriction and increased flow resistance. Consequently, the conventional understanding of CSF as a mere angiographic finding has been superseded by a more nuanced and comprehensive perspective, recognizing it as a pathology of microvascular, endothelial, and inflammatory origin [7,19,20].

Hematological factors have also been implicated in the pathogenesis of CSF. While some studies have reported elevated platelet counts, mean platelet volume, and aggregation indices in patients with CSF, our findings showed no statistically significant differences in platelet counts or other complete blood count parameters between groups. Similarly, the platelet-to-lymphocyte ratio, suggested as a predictive marker in other studies, did not differ significantly in our cohort. These discrepancies may be due to the retrospective design of this study and the inclusion of patients with acute symptoms.

Castelli risk indices have previously been described as independent risk factors for CSF. Nevertheless, we found no statistically significant differences in these indices between the patient and control groups. This inconsistency may result from differences in patient characteristics or timing of measurements. Further studies with more homogeneous populations may provide clearer insights.

In conclusion, our study demonstrated that readily accessible inflammatory markers, such as CRP and the CRP/albumin ratio, are significantly associated with CSF. These parameters could aid in identifying CSF in patients presenting to the emergency department with recurrent chest pain. Furthermore, the higher prevalence of Cx dominance in the CSF group suggests that coronary dominance patterns may influence coronary flow dynamics. While several other laboratory indices did not reach statistical significance in our analysis, this may be due to the acute nature of our patients’ presentations. Prospective, multicenter studies with broader inclusion criteria are needed to validate and expand upon these findings.

### Limitations

It is imperative to acknowledge the limitations of this study:

1. Single-Center and Retrospective Design: As a retrospective analysis conducted at a single institution, the results may not be generalizable to broader populations. Selection bias cannot be excluded.
2. Acute Patient Population: The study population consisted of patients presenting acutely to the emergency department, which may have affected the accuracy of certain indices such as the triglyceride/glucose index and Castelli risk scores.
3. Lack of Long-Term Follow-Up: Clinical outcomes such as recurrent hospital admissions, arrhythmia development, or major adverse cardiac events and prognosis were not tracked, limiting our ability to assess the prognostic value of the studied parameters.
4. Limited Sample Size: Although statistically powered for primary endpoints, a larger sample size would be beneficial for detecting more subtle associations between CSF and various laboratory or angiographic parameters.
5. Absence of Advanced Imaging or Functional Testing: Tools such as intravascular ultrasound (IVUS) or coronary physiology tests (e.g., coronary flow reserve), which could provide more detailed insight into endothelial and microvascular function, were not utilized.

Future prospective, multicenter studies with longer follow-up and broader patient populations are warranted to validate and expand upon these findings.

## 5. Conclusions

Patients with CSF frequently present to emergency departments with recurrent chest pain, significantly impairing their quality of life. CSF should not be considered merely an angiographic finding; rather, it is a clinically relevant condition that may lead to malignant arrhythmias and sudden cardiac death.

Our study demonstrates that inflammatory markers—particularly CRP and the CRP/albumin ratio—are significantly elevated in patients with CSF and may serve as valuable indicators in identifying high-risk individuals, especially in the acute care setting. Additionally, the observed association between coronary dominance patterns and CSF, particularly the increased prevalence of Cx dominance in the patient group, may offer further insights into the pathophysiological mechanisms of CSF.

While some other metabolic and hematologic indices (e.g., atherogenic index, triglyceride/glucose index, Castelli indices) did not show significant associations in our cohort, this may reflect the specific characteristics of our patient population. These findings underscore the potential clinical utility of simple, cost-effective laboratory parameters in predicting CSF and highlight the need for further investigation.

## Figures and Tables

**Table 1:** Demographic and clinical characteristics of patients.

	Control Group (*n* = 108)	Patient Group (*n* = 107)	Total (*n* = 215)	*p* Value
Gender				0.247 ^1^
Male	47 (43.5)	55 (51.4)	102 (47.4)	
Female	61 (56.5)	52 (48.6)	113 (52.6)	
Age, (years)	59.9 ± 10.4	58.9 ± 10.3	59.4 ± 10.3	0.434 ^2^
Hypertension	57 (52.8)	58 (54.2)	115 (53.2)	0.834 ^1^
Diabetes	27 (25.0)	32 (29.9)	59 (27.4)	0.420 ^1^
Hyperlipidemia	56 (51.9)	64 (59.8)	120 (55.8)	0.240 ^1^
Dominance				<0.001 ^1^
Cx	38 (35.2)	82 (76.6)	120 (55.8)	
RCA	70 (64.8)	25 (23.4)	95 (44.2)	
Slow Current Presence				-
Cx	-	64 (59.8)	64 (59.8)	
RCA	-	65 (60.7)	65 (60.7)	
LAD	-	94 (87.9)	94 (87.9)	

^1^ Chi-square test; ^2^ *t* test.

**Table 2:** Laboratory characteristics of patients.

	Control Group (*n* = 108)	Patient Group *(n* = 107)	Total *(n* = 215)	*p* Value
Total Cholesterol mg/dL *	203.4 ± 44.1	203.5 ± 47.8	203.4 ± 45.8	0.985 ^1^
LDL mg/dL *	117.9 ± 38.0	114.9 ± 39.5	116.5 ± 38.7	0.573 ^1^
HDL mg/dL *	52.5 ± 14.4	49.8 ± 11.3	51.1 ± 12.9	0.132 ^1^
TG mg/dL *	170.3 ± 105.1	192.6 ± 155.7	181.3 ± 132.8	0.219 ^1^
Glucose mg/dL *	117.6 ± 39.7	123.7 ± 54.4	120.6 ± 47.6	0.348 ^1^
Albumin g/L *	40.6 ± 3.3	40.1 ± 3.1	40.3 ± 3.2	0.244 ^1^
Uric Acid mg/dL *	5.6 ± 1.6	5.8 ± 1.7	5.7 ± 1.7	0.501 ^1^
Creatinine mg/dL *	0.9 ± 0.4	0.9 ± 0.5	0.9 ± 0.5	0.629 ^1^
Hemoglobin g/dL *	12.9 ± 1.4	13.6 ± 1.6	13.2 ± 1.5	0.002 ^1^
Neutrophil 10^3^/µL *	4.5 ± 1.6	4.9 ± 1.9	4.7 ± 1.7	0.060 ^1^
Lymphocyte10^3^/µL *	2.1 ± 0.8	2.3 ± 0.7	2.2 ± 0.7	0.097 ^1^
Monocyte 10^3^/µL *	0.5 ± 0.1	0.6 ± 0.2	0.6 ± 0.2	0.135 ^1^
Platelet 10^3^/µL *	257.2 ± 79.4	266.7 ± 68.6	261.9 ± 74.2	0.347 ^1^
CRP mg/L **	0 (0–200.0)	3.2 (3.1–144.0)	3.1 (0–200.0)	<0.001 ^2^
WBC 10^3^/µL **	7.3 (4.0–115.0)	7.7 (4.6–16.4)	7.6 (4.0–115.0)	0.011 ^2^
Troponin ng/L **	3.4 (2.3–671.0)	3.4 (2.1–2803.6)	3.4 (2.1–2803.6)	0.300 ^2^

^1^ *t* test; ^2^ Mann–Whitney U test; * mean ± standard deviation; ** median (min–max).

**Table 3:** Index values of patients.

	Control Group (*n* = 108)	Patient Group (*n* = 107)	Total (*n* = 215)	*p* Value
CRP/Albumin *	0 (0–5.6)	0.1 (0.1–3.7)	0.1 (0–5.6)	*p* < 0.001 ^1^
Uric Acid/Albumin *	0.1 (0.1–0.3)	0.1 (0.1–0.3)	0.1 (0.1–0.3)	0.507 ^1^
TG/Glucose **	4.8 ± 0.3	4.9 ± 0.4	4.8 ± 0.3	0.185 ^2^
Plasma Atherogenic Index **	0.1 ± 0.3	0.2 ± 0.3	0.1 ± 0.3	0.131 ^2^
Castelli Risk Index ^1,^*	4.0 (1.4–7.3)	4.2 (2.0–13.3)	4.1 (1.4–13.3)	0.390 ^1^
Castelli Risk Index ^2,^*	2.3 (0.3–5.0)	2.3 (0.4–5.3)	2.3 (0.3–5.3)	0.887 ^1^

^1^ Mann–Whitney U test; ^2^ *t* test; * median (min–max); ** mean ± standard deviation.

## Data Availability

Due to privacy concerns, the data presented in this study are available from the corresponding author upon request.
